# A crucial role for β2 integrins in podosome formation, dynamics and Toll-like-receptor-signaled disassembly in dendritic cells

**DOI:** 10.1242/jcs.151167

**Published:** 2014-10-01

**Authors:** Christian Gawden-Bone, Michele A. West, Vicky L. Morrison, Alexander J. Edgar, Sarah J. McMillan, Brian D. Dill, Matthias Trost, Alan Prescott, Susanna C. Fagerholm, Colin Watts

**Affiliations:** 1 Division of Cell Biology and Immunology, College of Life Sciences, University of Dundee, Dundee DD1 5EH, UK; 2 University of Dundee, Ninewells Hospital and Medical School, Dundee DD1 9SY, UK; 3 MRC Protein Phosphorylation and Ubiquitylation Unit, College of Life Sciences, University of Dundee, Dundee DD1 5EH, UK

**Keywords:** Podosomes, Dendritic cells, Integrins

## Abstract

The dynamic properties of podosomes, their ability to degrade the underlying matrix and their modulation by Toll-like receptor (TLR) signaling in dendritic cells (DCs) suggests they have an important role in migration. Integrins are thought to participate in formation and dynamics of podosomes but the multiplicity of integrins in podosomes has made this difficult to assess. We report that murine DCs that lack β2 integrins fail to form podosomes. Re-expression of β2 integrins restored podosomes but not when the membrane proximal or distal NPxF motifs, or when an intervening triplet of threonine residues were mutated. We show that β2 integrins are remarkably long-lived in podosome clusters and form a persistent framework that hosts multiple actin-core-formation events at the same or adjacent sites. When β2 integrin amino acid residues 745 or 756 were mutated from Ser to Ala, podosomes became resistant to dissolution mediated through TLR signaling. TLR signaling did not detectably modulate phosphorylation at these sites but mutation of either residue to phospho-mimetic Asp increased β2 integrin turnover in podosomes, indicating that phosphorylation at one or both sites establishes permissive conditions for TLR-signaled podosome disassembly.

## INTRODUCTION

Podosomes are actin-rich structures found on the ventral plasma membrane of macrophages, dendritic cells (DCs), osteoclasts and endothelial cells. Unlike other adhesive structures podosomes are able to degrade the underlying matrix, a property that has led to frequent comparisons with invadopodia of transformed cells (Gimona et al., 2008; Linder, 2007; Murphy and Courtneidge, 2011). Recent studies have shown that podosomes in DCs and other cells exert force on the underlying substrate that, together with their matrix-degrading ability, can result in protrusion into the underlying substrate (Collin et al., 2008; Gawden-Bone et al., 2010; Labernadie et al., 2010; Schachtner et al., 2013; van den Dries et al., 2013a). Podosomes feature two structural domains, an actin-rich core and a surrounding ‘ring’ region that contains actin-binding and -adhesive proteins, including integrins and integrin regulatory proteins such as talin (Chou et al., 2006), kindlin-3 (Schmidt et al., 2011) and paxillin (Badowski et al., 2008). A more diffuse network of actin – which also contains myosin II – is found between podosomal actin cores and, by generating tension, contributes to the oscillatory, probing behavior of the actin core units (Destaing et al., 2003; Gawden-Bone et al., 2010; Luxenburg et al., 2007; van den Dries et al., 2013b; Yu et al., 2013). In DCs, podosomes assemble into clusters, where rings merge to form a honeycomb-like structure around actin cores (Burns et al., 2004; West et al., 2004).

DCs are tissue-resident, pathogen-sensing cells that collect, process and present antigen following migration to lymphoid organs. Because tissue-located DCs must cross several tissue and matrix barriers to reach lymphoid organs it has been proposed that podosomes constitute an important part of their migratory apparatus. We and others have shown that DC maturation stimuli, including Toll-like receptor (TLR) ligands and prostaglandin E2 (PGE2), trigger rapid podosome disassembly in mouse and human DCs (van Helden et al., 2006; West et al., 2004).

Although integrins are recognized as crucial components of podosomes, the precise features of integrins that regulate podosome formation, function and turnover have not been established because in most cells multiple integrins are found in podosomes (reviewed in Dovas and Cox, 2011; Linder, 2009). In a recent study, ablation of β1, β2 and β3 integrins was necessary to eliminate podosomes in osteoclasts (Schmidt et al., 2011). The cytoplasmic tail of β2 integrin (encoded by *Itgb2*) comprises several regions that are important for integrin function, including a membrane proximal NPxF domain important for talin recruitment and a distal NPxF domain (Moser et al., 2009b) that, in combination with a triplet of preceding threonine residues (Thr758–760 in human β2 integrin; Thr759–761 in murine β2 integrin), binds kindlin-3 (also known as FERMT3) (Gahmberg et al., 2009; Morrison et al., 2013; Moser et al., 2009a). Recently, we have shown that T cells in which Thr758–760 had been mutated to Ala758–760 have reduced adhesion and defective homing *in vivo* (Morrison et al., 2013). Additional putative regulatory residues in the β2 integrin cytoplasmic tail including Ser745 and Ser756 have been described (Fagerholm et al., 2005; Fagerholm et al., 2002; Perez et al., 2003). So far, the contribution and role of these β2 integrin tail motifs in regard to podosome structure and function has not been directly investigated, not least because multiple integrins may have a role in podosome formation (reviewed in Linder, 2009). We show here that murine DCs that lacking β2 integrins fail to form podosomes, a finding that has allowed for the first time some probing of the features of integrins that control podosome formation and dynamics as well as TLR-signaled disassembly.

## RESULTS

### β2 integrins are essential for podosome formation in cultured and *ex vivo* murine DCs

**Fig. 1.**
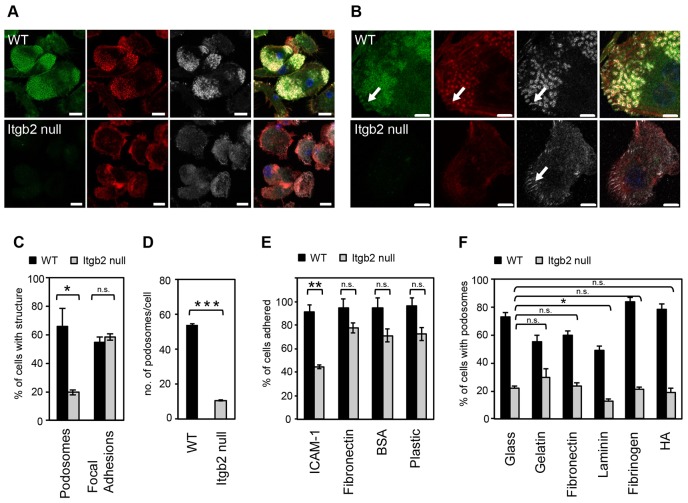
**β2-integrin-null DCs are podosome deficient.** Wild type (WT) and Itgb2-null SDCs plated on glass coverslips were fixed and stained for β2 integrin (green; FITC), F-actin (red; Alexa-Fluor-555) and vinculin (grey; Alexa-Fluor-633). (A) WT cells contained podosome clusters with clear actin cores, and β2 integrin- and vinculin-rich podosome rings and/or plaques. Itgb2-null DCs adhered but did not form podosomes. (B) WT and Itgb2-null DCs, can both form focal adhesions (white arrows). Single optical sections of 0.7 µm, taken at the ventral surface of the cells were acquired using Zen 2009 software on a Carl Zeiss 700 confocal laser-scanning microscope with a 100x Plan Apochromat/NA 1.46 oil immersion objective. Scale bars: 10 µm (A), 5 µm (B). (C) Percentage of integrin-null cells containing podosomes confirms the dramatic lack of podosomes compared to WT DCs (*P = 0.01, unpaired *t*-test), whereas the percentage of cells containing focal adhesions is normal. (D) Individual podosomes were also counted in WT and Itgb2-null SDCs and the results demonstrate that individual Itgb2-null cells have less podosomes compared with WT (29–57 cells scored per sample, error bars are s.e.m. for triplicate biological samples, ***P<0.001, unpaired *t*-test). (E) DCs were plated for 75 min on substrates as indicated and the % of adherent cells assessed after washing. Only when Itgb2-null cells were plated on β2 substrate, ICAM-1, was there a significant reduction in adhesion (**P = 0.002, unpaired *t*-test). (F) DCs were plated for 2 hours onto coverslips coated with gelatin, fibronectin, laminin or fibrinogen (all at 10 µg/ml), or with HA (100 µg/ml), and cells with podosomes quantified after staining for F-actin and vinculin. There was no significant rescue of podosomes in Itgb2-null DCs when plated on the various substrates compared with plating on glass alone, except when cells were incubated on laminin, in which case podosome levels were reduced rather than rescued (paired *t*-tests).

PCR analysis, fluorescence-activated cell sorting (FACS) and immunofluorescence revealed that murine DCs expressed β integrin chains 1, 2, 3 and 5, αM, αX and αL but not αD (supplementary material Fig. S1 and data not shown). At the protein level, positive staining in podosome rings of DCs was seen for αM, αX, α6 and, consistent with earlier studies in human DCs, β2 (Burns et al., 2004; van den Dries et al., 2013b). To address the possibility that β2 integrins mediate the adhesion necessary for podosome formation we expanded DCs from bone marrow and spleen of β2-integrin-null mice and wild type DCs. Remarkably, DCs from β2-integrin-null mice showed a striking reduction in podosome-containing cells when compared to littermate wild type cells (Fig. 1A,C; supplementary material Fig. S2A). Moreover, the number of podosomes per cell was four to five times lower in the few β2-integrin-null cells that still formed podosomes (Fig. 1D). To establish the authenticity of the DCs from β2-integrin-null mice we measured the expression of characteristic DC markers. As expected, the β2-chain-dependent integrins CD11c and CD11b were not expressed. Cell surface markers, such as MHC class II, CD54, CD86, CD274 and others were, however, expressed on the surface of β2-integrin-null DC and their expression was increased by LPS-driven maturation, confirming that the cells are, indeed, DCs (supplementary material Fig. S1C and data not shown).

Importantly, focal adhesions were not significantly affected by loss of β2 integrins, suggesting that the defect in podosome formation was not simply due to failure to adhere to the substrate (Fig. 1B,C). To address this more directly, we plated β2-integrin-null DCs and wild type DCs on various surfaces and, 75 minutes later, removed non-adherent cells by washing. As shown in Fig. 1E, both cell types adhered equally well to plastic, BSA or fibronectin. Adherence of β2-integrin-null DCs to the β2-integrin ligand ICAM1 was substantially reduced, as expected. FACS analysis of β2-integrin-null DCs showed that β1 and β3 integrins were still expressed at levels comparable to those in wild type cells (supplementary material Fig. S1A). β5 integrins were not detected by FACS in either cell type but were prominent in focal adhesions in both wild type and β2-integrin-null DCs (supplementary material Fig. S1B). We asked whether podosomes could be rescued by plating the β2-integrin-null cells on dishes coated with β1 and β3 integrin ligands, including gelatin, fibronectin, laminin, fibrinogen or hyaluronic acid, the latter being a CD44 ligand thought to be required for podosome stability (Chabadel et al., 2007). These matrices supported podosomes in 50–85% of wild type cells but in only 15–30% of β2-integrin-null cells, depending on the substrate (Fig. 1F). Importantly, these alternative matrices were intact beneath the cells, ruling out the possibility that they had been degraded and were, therefore, not available anymore (data not shown). Furthermore, increasing the time that cells interacted with the substrate (supplementary material Fig. S2B) or addition of the integrin activator MnCl_2_ (supplementary material Fig. S2C) did not rescue podosome formation in β2-integrin-null cells.

**Fig. 2.**
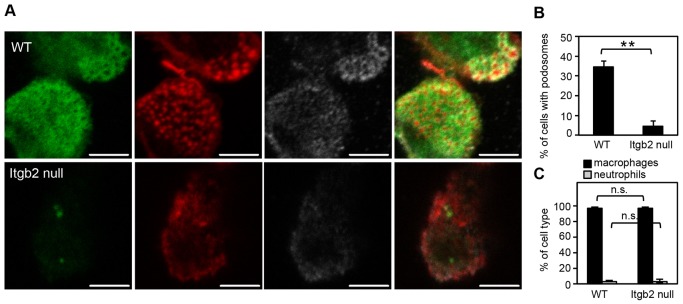
***Ex vivo* cells that lack β2 integrin have podosome formation defects.** (A) Resident lung cells (>95% alveolar macrophages) collected from wild type (WT) or Itgb2-null mice by bronchoalveolar lavage were plated on coverslips and stained for β2 integrin (green; FITC), F-actin (red; Alexa-Fluor 555) and vinculin (grey; Alexa-Fluor 633). Images were acquired as described for Fig. 1. Scale bars: 5 µm. (B) Adherent cells were scrutinized for podosome formation using systematic scanning of the coverslips. Quantification of the percentage of cells with podosomes indicated a strong defect in podosome formation in lung-derived cells (**P = 0.001, unpaired *t*-test). (C) Cellular composition of bronchoalveolar lavage in WT and Itgb2-null mice was determined morphologically by differential counts of DiffQuik-stained cytospin preparations.

To test whether the loss of individual β integrin α chains affected podosome formation we cultured DCs from mice lacking individual αL, αM or αX chains but found no significant effect on the number of cells containing podosomes (supplementary material Fig. S2D). Thus, podosome formation in DCs does depend on β2 integrins but does not need a specific integrin α chain. β2 integrins were also crucial for podosome formation in *ex vivo* myeloid-/monocyte-derived cell populations because we observed fewer podosomes forming in cells that had been isolated from lungs of β2-integrin-null mice compared with those isolated from wild type mice (Fig. 2A,B). Loss of β2 integrin did not change the cellular composition of the lung lavage; of both wild type and β2-integrin-null mice more than 95% of cells were alveolar macrophages (Fig. 2C). In contrast to DCs, and in agreement with an earlier study (Schmidt et al., 2011), osteoclasts from β2-integrin-null mice were still able to form belt-like podosome structures (supplementary material Fig. S2E).

### Divergence of integrin β2 and actin-core lifetimes in DC podosomes

**Fig. 3.**
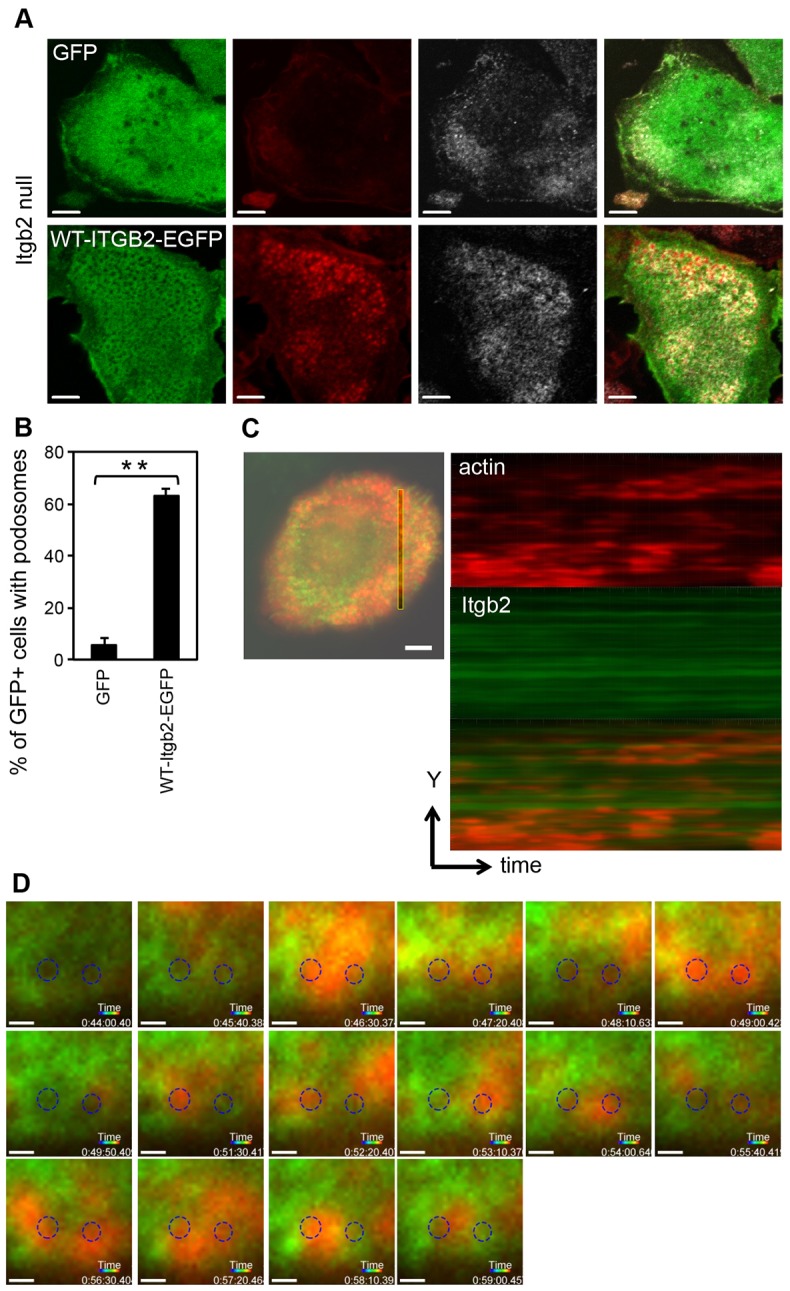
**Retroviral expression of Itgb2-EGFP rescues podosomes in Itgb2-null DCs.** Itgb2-null BMDCs were infected with retrovirus encoding a Itgb2-EGFP fusion protein, or GFP alone as a control. (A) The infected DCs were allowed to adhere to glass, then fixed and stained for F-actin (red; Alexa Fluor-555) and vinculin (grey; Alexa Fluor-633). Images were acquired using a Zeiss LSM700 as in Fig. 1. Scale bars: 5 µm. (B) Percentage of infected (GFP^+^) cells that contain podosomes, demonstrating significant reconstitution of podosomes with WT-β2 integrin compared to GFP alone (**P = 0.007, paired *t*-test). (C) Retrovirus encoding both Itgb2-EGFP and Lifeact-mCherry was used to infect Itgb2-null DCs. The cells were plated onto glass dishes and images collected every 10 seconds at 37°C using a Nikon Eclipse Ti TIRF microscope with an ApoTIRF 100x/NA1.49 objective as in Materials and Methods. A section through a podosome cluster was selected and Imaris software used to convert time to display on the *z*-axis, to generate a kymograph (Itgb2, green; actin, red; sequence represents 491 seconds), revealing the transient nature of the actin cores compared with the long-lived Itgb2-EGFP. To measure the lifetime of podosome cores, 682 podosomes were observed in six cells from at least three separate experiments. Scale bar: 5 µm. (D) A series of selected images (50-second intervals) shows that stable EGFP-labelled Itgb2 structures can support the reoccurrence of podosome cores in the same location after long periods of time (circled areas; images were cropped and circles were added to aligned layers using Photoshop CS5); ∼17±3% of sites (810 observed podosomes in six plaques from three experiments) hosted the return of actin cores. Scale bars: 0.5 µm.

We took advantage of the specific requirement for β2 integrins in murine DCs podosomes to analyze wild type and mutant integrins without interference from endogenous integrins. As shown in Fig. 3A,B, expression of EGFP-tagged β2 integrin (Itgb2-EGFP) in β2-integrin-null cells restored podosomes, whereas the expression of a control construct expressing only GFP did not. To simultaneously image F-actin with β2 integrins, we infected DCs with a bi-cistronic retroviral vector encoding Itgb2-EGFP and Lifeact-mCherry, and observed live cells by using total internal reflection fluorescence microscopy. From kymograph (Fig. 3C) measurements (West et al., 2008), the average lifetime of actin cores was 196±23 seconds, which is in good agreement with previous publications describing the use of actin-EGFP (Evans et al., 2003; West et al., 2008). However, under the same conditions we were unable to measure integrin lifetime in podosome rings because the EGFP-labelled integrin was already present prior to actin core appearance and persisted beyond the time available before photo-bleaching occurred (Fig. 3C). This indicated that integrins in podosome rings are surprisingly long lived.

To explore this further we expressed EGFP-tagged kindlin-3 and Lifeact-mCherry in wild type DCs. Similar to Itgb2-EGFP, EGFP-Kindlin-3 was distributed at the membrane prior to the formation of actin cores and outlasted them, persisting in a honeycomb-like network similar to that generated by Itgb2-EGFP (supplementary material Fig. S3; Movie 1). This suggests that integrins are activated and stable over the lifetime of a podosome cluster that contains multiple actin cores. Importantly, ∼17±3% of sites within the stable integrin array hosted the return of actin cores to the same location with more actin cores becoming established directly adjacent to the previous core (Fig. 3C,D; supplementary material Movie 2).

**Fig. 4.**
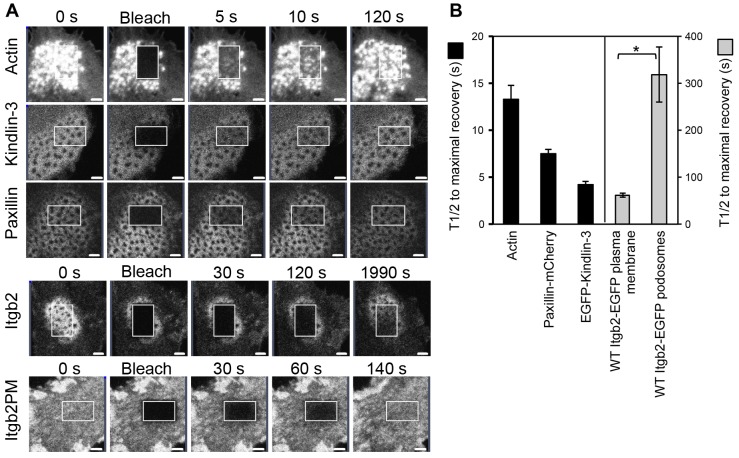
**FRAP analysis of Itgb2-EGFP lifetime in podosomes.** BMDCs were infected with retroviruses for expression of actin-EGFP, EGFP-kindlin3, paxillin-mCherry or Itgb2-EGFP (Itgb2-null cells). The cells were plated into glass-bottomed dishes and an area within a podosome cluster was photobleached using a Zeiss LSM700 confocal microscope as in described in Materials and Methods. Cells were then imaged over time to follow fluorescence recovery (A). For the Itgb2-EGFP-expressing cells an additional area of cell was also bleached to assess integrin turnover outside of podosomes (Itgb2PM). The fluorescence recovery in podosomes of actin-EGFP, EGFP-kindlin-3, paxillin-mCherry and Itgb2-EGFP in the plasma membrane were all relatively rapid compared to the recovery of Itgb2-EGFP in podosomes. (B) Recovery curves for each tagged protein were normalized for comparison and mean *t*_1/2_ values calculated, each from three independent experiments (three individual BMDCs cultures and viral infections), analyzing a minimum of ten cells per experiment (*P* = 0.04, paired *t*-test, comparing β2 integrin in podosomes versus plasma membrane). Scale bars: 2 µm.

To further test the dynamics of integrins, integrin-associated proteins and actin in podosome clusters we performed fluorescence recovery after photo-bleaching (FRAP) experiments. The half-time to recovery after photo-bleaching of the wild type Itgb2-EGFP signal in a podosome cluster was 319±59 seconds (Fig. 4A,B), more than five times slower than FRAP recovery times for integrins outside of podosome plaques (61.8±4 seconds; Fig. 4A,B). Moreover, the immobile fraction, i.e. the proportion of the original Itgb2-EGFP signal that fails to recover following FRAP, was greater in podosomes (data not shown). As shown in Fig. 4, the slow turnover of integrins in podosomes, measured by FRAP, contrasted with much faster turnover of actin-EGFP (13.2±1.5 seconds), EGFP-kindlin-3 (4.2±0.2 seconds) and paxillin-mCherry (7.5±0.4 seconds) in podosomes. These results show that integrins found in podosome clusters are remarkably persistent compared with the actin cores and, indeed, some integrin-associated proteins.

### Both NPxF motifs and Thr758–760 in β2 integrin are required for podosome formation

**Fig. 5.**
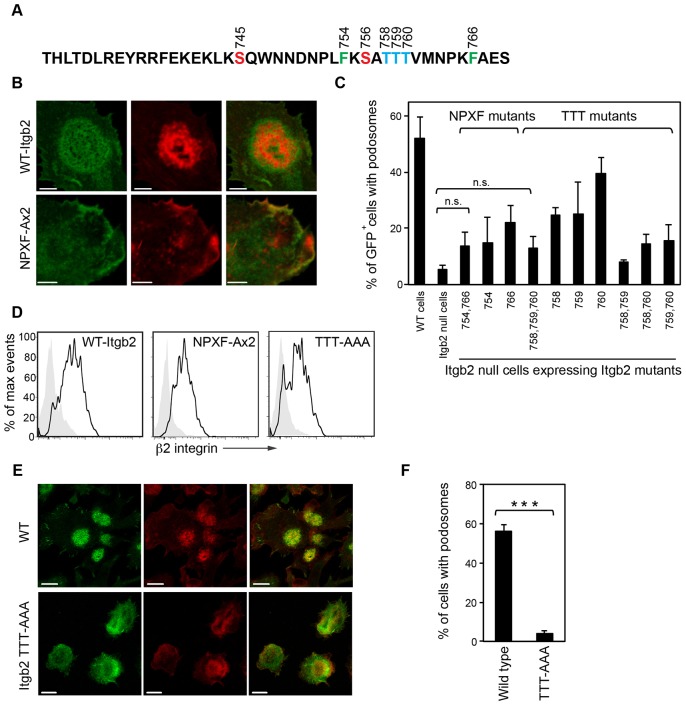
**Mutation of key residues in the cytoplasmic tail of Itgb2 and their effect on podosome formation.** (A) Amino acid sequence of the cytoplasmic tail of the mouse β2 integrin with residues of interest highlighted; Ser745, Ser756 (red), Thr758, Thr 759, Thr 760 (TTT; blue), Phe754 and Phe766 (green). Amino acid positions are based on the human β2 integrin. (B) Itgb2-null cells were reconstituted with either wild type Itgb2-EGFP (WT-Itgb2) or NPXF-Ax2-Itgb2-EGFP (NPXF-Ax2) by retroviral infection (green). After fixation, the cells were stained for F-actin (red; Alexa-Fluor-555). The Itgb2-NPXF-A mutant was unable to rescue podosome formation even though it localized to the plasma membrane. (C) Percentage of podosomes in control WT cells or Itgb2-null cells reconstituted with GFP alone or with Itgb2 constructs containing the following cytoplasmic tail mutations as indicated. NPxF-Ax2: F754A, F766A. TTT-AAA: T758A, T759A, T760A, and double and triple combinations thereof. Data are from 100–150 GFP-positive cells per sample. Podosomes were not significantly reconstituted in Itgb2-null cells expressing the NPXF-Ax2 or TTT-AAA mutants compared to GFP alone (paired *t*-tests). (D) Cell surface expression of the WT-Itgb2 and NPxF-Ax2 and TTT-AAA mutants in the infected cells was confirmed by flow cytometry using an anti-β2 integrin antibody. (E) BMDCs cultured from Itgb2WT or Itgb2 TTT-AAA β2 cytoplasmic tail knock-in mice were stained for β2 integrin (green; FITC) and F-actin (red; Alexa-Fluor-555). Scale bars: 5 µm (B) and 10 µm (E). (F) Percentage of cells with podosomes indicates a loss of the podosome phenotype in the TTT-AAA knock-in DCs (***P<0.001, unpaired *t*-test). Images were acquired using a Zeiss LSM700, as in Fig. 1.

Having demonstrated the requirement for β2 integrins in podosome formation we speculated that the cytoplasmic tail plays a key role in this process and also potentially in podosome dissolution driven by TLR signaling or other stimuli. To test this we generated a series of integrin constructs with mutations in the cytoplasmic domain (Fig. 5A) and expressed these by retroviral infection in β2-integrin-null DCs. Where the Phe residues in both NPxF domains were mutated to Ala (Phe754Ala and Phe766Ala; referring to amino acid residue positions in human β2 integrin throughout), mutant β2 integrins reached the plasma membrane but failed to induce podosome formation (Fig. 5B,C). Individual proximal NPxA (F754A) and distal NPxA (F766A) β2 integrin variants, which had been predicted to interfere with talin and kindlin-3 binding, respectively, were also unable to support podosome formation (Fig. 5C). Failure to reconstitute podosomes was not owing to reduced expression of some integrin mutants because, when we gated on retrovirally infected GFP-positive DCs and then directly measured cell surface β2 integrin levels, they were comparable to wild type (Fig. 5D and data not shown).

Previous data identified a triplet of Thr residues (Thr758–760) within the β2 cytoplasmic tail as crucial for inside-out activation of integrins in T cells and, together with the membrane distal NPxF motif, for kindlin-3 binding (Fagerholm et al., 2005; Nurmi et al., 2007). We, therefore, examined DCs from a newly established mouse strain, in which Thr758–760 are mutated to alanine (TTT/AAA-β2-integrin knock-in mouse) (Morrison et al., 2013). As shown in Fig. 5E,F, DCs derived from these mice were unable to form podosomes. A comparable shortfall in podosomes was observed when β2-null DCs were reconstituted with the same triple mutant and a more modest deficit was seen when single Thr758Ala and Thr759Ala mutants were expressed (Fig. 5C, data not shown). Taken together, these results show that podosome formation in murine DCs is crucially dependent on three key motifs within the β2 integrin cytoplasmic tail needed for integrin function and which have been shown to be necessary to engage with talin, kindlin-3 and other integrin partners.

### Ser745 and Ser756 are crucial for acute LPS-signaled podosome dissolution

**Fig. 6.**
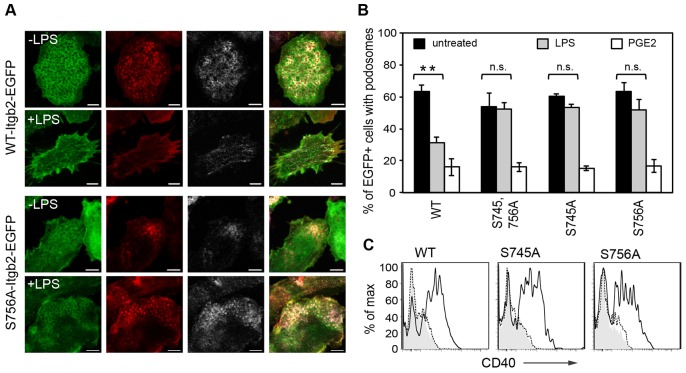
**Mutation of Ser756 to Ala blocks acute LPS-stimulated podosome loss.** (A) Itgb2-null BMDCs were infected with retroviral constructs expressing WT-Itgb2-EGFP or S756A-Itgb2-EGFP (green). Cells were treated as indicated with (+LPS) or without (–LPS) 50 ng/ml LPS for 30 minutes, then fixed and stained to visualize F-actin (red; Alexa-Fluor-555) and α-actinin 4 (grey; Alexa-Fluor-633). Podosomes reconstituted with the S756A-Itgb2-EGFP mutant were resistant to the LPS-stimulated disassembly seen for WT-Itgb2-EGFP (A; green). Images were acquired using a Zeiss LSM700, as in Fig. 1. Scale bars: 5 µm. (B) Percentage of EGFP+ cells that contain podosomes when reconstituted with Itgb2-EGFP constructs containing double or single Ser to Ala mutations (S745A and S756A, or either S745A or S756A) with or without treatment with LPS (50 ng/ml) or prostaglandinE2 (10 µg/ml) for 30 minutes, before fixation and staining as above. LPS-driven podosome loss, though significant (**P = 0.005) for WT-Itgb2-EGFP, was not significant in cells expressing the single S745A or S756A β2 mutants (paired *t*-tests). (C) CD40 expression in cells expressing WT, S745A or S756A Itgb2 was assessed by flow cytometry in control cells (dashed line) and after 20 hours of LPS treatment (solid line).

In T cells at least part of the functionality of the Thr triplet discussed above is due to phosphorylation of Thr758 (Nurmi et al., 2007). Phosphorylation of Ser745 and Ser756 has also been demonstrated, although there are only limited data available on which physiological stimuli are relevant and what impact this phosphorylation might have (Fagerholm et al., 2002; Lim et al., 2011; Perez et al., 2003). The significance of these residues regarding integrin function in DCs has not been explored. We introduced Ser-to-Ala mutations at one or both residues (Ser745, Ser756) of Itgb2-EGFP and expressed the construct in β2-integrin-null DCs. In contrast to the mutants described above, β2-integrin-null BMDCs expressing these mutations formed podosomes normally (Fig. 6A). We and others have shown that podosomes in DCs are acutely destabilized by TLR signaling as well as by other stimuli, such as PGE2 (van Helden et al., 2006; van Helden et al., 2008; West et al., 2004; West et al., 2008). As expected, after 30 minutes of treatment with LPS (Fig. 6A,B) or PGE2 (Fig. 6B), β2-integrin-null DCs that were reconstituted with Itgb2-EGFP showed a substantial reduction in the number of cells with podosomes. Although DCs that had been reconstituted with β2 integrin carrying single or double S745A and S756A mutations disassembled their podosomes when challenged with PGE2, podosomes persisted when cells were challenged with LPS, (Fig. 6A,B). DCs expressing these mutants were still responsive to LPS because the activation markers CD40 and class II MHC were upregulated normally (Fig. 6C and data not shown). This striking result raised the possibility that phosphorylation of Ser745 or Ser756 is involved in LPS-signaled podosome dissolution. To assess this we stimulated wild type BMDCs with LPS and immunoprecipitated β2 integrins prior to tryptic digestion and analysis by mass spectrometry. Although we were not able to detect phosphorylated Ser in the peptide containing Ser745, we identified a singly phosphorylated form of the peptide containing Ser756 and the Thr triplet (Thr758–760) (supplementary material Fig. S4). Analysis of the fragmentation spectrum identified the phosphorylation site to be at Ser756. However, stimulation with LPS did not increase the amount of this phosphorylated peptide relative to the pool of β2 integrin. In contrast, treatment with PMA boosted phosphorylation of this site tenfold, consistent with earlier results in T cells (supplementary material Fig. S4A,C) (Fagerholm et al., 2002).

**Fig. 7.**
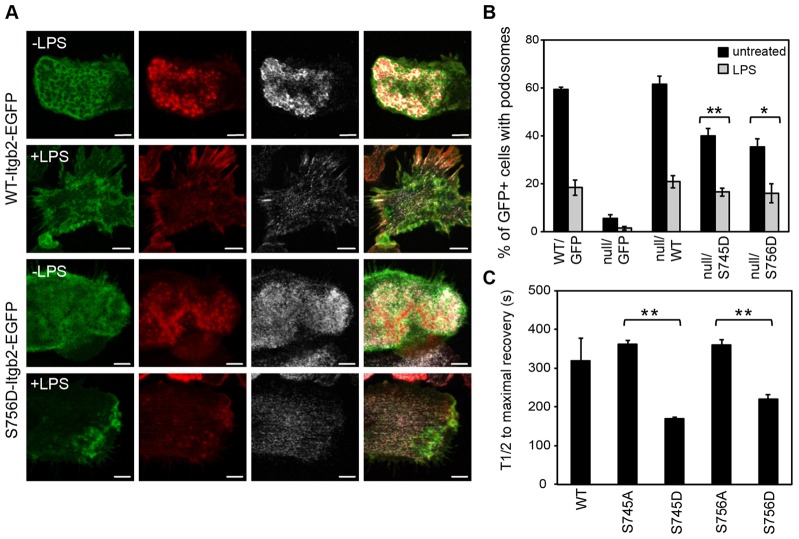
**Mutation of Ser756 to Asp rescues acute LPS-driven podosome loss.** (A) Itgb2-null BMDCs were infected with retroviruses containing either the WT-Itgb2-EGFP or the S756D-Itgb2-EGFP mutant constructs (green EGFP staining) and treated with (+LPS) or without (–LPS) 50 ng/ml LPS for 30 minutes before fixation and then stained to visualize F-actin (red) and α-actinin 4 (grey). Images were acquired using a Zeiss LSM700, as described for Fig. 1. The images show that podosomes formed normally in S756D-Itgb2-EGFP expressing cells and that LPS induced podosome dissolution. Scale bars: 5 µm. (B) Percentages of infected (GFP positive) cells showing podosomes when reconstituted with empty vector (pBMN-I-GFP), or constructs for WT β2 integrin or the S745D and S756D mutants, with or without LPS treatment were quantitated. Podosomes in cells expressing the S745D and S756D mutants were responsive to LPS (**P = 0.001 and *P = 0.014, unpaired *t*-tests). (C) Itgb2-null BMDCs expressing EGFP fusion proteins of WT, S745A, S745D, S756A or S756D-Itgb2 were cultured in glass bottom dishes for FRAP analysis, as in Fig. 4. Cells expressing Itgb2-EGFP in a typical honeycomb shaped podosome ring pattern in the ventral plasma membrane were selected for photobleaching and the half-life for fluorescence recovery measured for each construct as in Materials and Methods (ten cells per experiment). S745D and S756D mutants show reduced half-life compared to corresponding Ala mutants (**P = 0.001 and **P = 0.006, respectively, paired *t*-test). Data were from three independent experiments from different bone marrow and virus preparations.

Although LPS did not detectably modulate phosphorylation at Ser756, the fact that its mutation to Ala blocked LPS-signaled podosome dissolution suggested that phosphorylation is, nonetheless, permissive for this effect. In this case, we reasoned that addition of a negative charge at these sites might change the properties of β2 integrins in podosomes. We, therefore, mutated Ser756 and Ser745 to Asp. β2-integrin-null cells that express these mutants still formed podosomes (Fig. 7A,B), albeit at somewhat reduced levels; and treatment with LPS triggered podosome dissolution similar to that observed in cells infected with wild type β2 integrin, reversing the effect of the Ala mutations (Fig. 7A,B). We then repeated our FRAP analysis comparing Itgb2-EGFP mutants carrying the Ser-to-Ala or the Ser-to-Asp mutations at Ser745 or Ser756. As shown in Fig. 7C, the Ser745Ala and Ser756Ala mutants showed a recovery time very similar to that of wild type (362±11 and 361±10 seconds respectively; compare with Fig. 4B), whereas the Ser745Asp and Ser756Asp mutants recovered approximately twice as fast (170±4 and 221±10 seconds respectively). Thus, β2 integrin that carries a negative charge at amino acid positions 756 and/or 745 is more mobile and exchangeable than its uncharged equivalent and might, therefore, have a lower affinity for the underlying substrate, for key cytoskeletal components or both. Even sub-stoichiometric phosphorylation at these sites, when combined with other TLR-signaled events, might critically compromise podosome stability.

## DISCUSSION

Although several integrins have been localized to podosomes, few studies have defined specific integrin requirements for podosome formation and dynamics. β1 and β3 integrins – rather than β2 integrin – have been more commonly identified at invadosome (podosome and invadopodia) sites (reviewed in Linder, 2009). For example, in MEFs and pre-osteoblastic cells, invadosome formation triggered by activation of Src was crucially dependent on β1A but not β3 integrin, even though the latter strongly colocalized with actin (Destaing et al., 2010). In human monocyte-derived macrophages, β1 rather than β2 integrin was found in 2D podosome rosettes and 3D podosome-like protrusions (Van Goethem et al., 2011). Some earlier studies have reported localization of β2 integrins to human DC podosomes (Burns et al., 2004; van den Dries et al., 2013b), but a strict requirement for β2 integrins has not been tested. Indeed, in murine osteoclasts podosomes and podosome-derived actin belts were still observed in doubly deficient β1-/β2-integrin-null mice, implying redundancy of multiple β integrin classes in this setting (Schmidt et al., 2011). Here, we show for the first time the requirement for β2 integrins for podosome formation. Because DCs that lack β2 integrins were still adherent and had normal focal adhesions, we were able to focus on integrin features specifically required for podosome formation, dynamics and modulation by TLR-signaling.

Reconstitution of β2-integrin-null cells with integrin mutants that lack kindlin-3- or talin-binding regions due to mutations in their NPxF motifs did not rescue podosome formation, indicating that podosome formation in DCs requires these key regulators of integrin activation and/or function. These data mirror a talin requirement for focal adhesion formation (Liu et al., 2011) and are in agreement with data showing that kindlin-3 is crucial for podosome formation in osteoclasts (Schmidt et al., 2011). Focal adhesions still formed in β2-integrin-null DCs – as did podosomes in osteoclasts – because, in contrast to DC podosomes, other β integrins with intact talin- and kindling-binding sites were able to substitute for β2 integrin. Our recent study, using a Thr758–760 TTT/AAA β2-integrin knock-in mouse, demonstrated the crucial importance of this motif for binding of kindlin-3 to β2 integrins, for strengthening of adhesion in T cells under shear flow and for homing of T cells *in vivo* (Morrison et al., 2013). We show here that DCs from these mice lack podosomes, which is likely to reflect the crucial importance of kindlin-3 binding to the β2 cytoplasmic tail. Taken together, our data highlight the crucial role of β2 integrins in DC podosomes and aligns the mechanism of integrin activation therein to kindlin-3-driven podosome formation in osteoclasts that, in contrast to DCs, have a redundant integrin requirement (Schmidt et al., 2011).

Further studies are needed to define why β2 integrin plays such a key role in podosome formation in DCs. It is striking that several sequence features distinguish β2 from other β integrins that are expressed in these cells. Other β chains (β1, β3, β5, β7) feature a membrane proximal NPxY motif rather than NPxF found in β2 integrin and – with the exception of β7 integrin – NxxY replaces the membrane distal NxxF motif in β2 integrin (Gahmberg et al., 2009). Tyr phosphorylation of NPxY and NxxY motifs has been shown to regulate binding of adaptors through their phosphotyrosine-binding (PTB) domains that vary in their sensitivity to the phosphorylated status of the Tyr residue (Legate and Fassler, 2009). Substitution of Phe for Tyr in β3 integrin perturbed the function of αIIbβ3 integrin in platelets, and led to impaired clot retraction and an *in vivo* bleeding phenotype, indicating a crucial role for Tyr phosphorylation – of at least β3 integrin – in this setting (Law et al., 1999). Clearly, adaptor binding to β2 integrin cannot be regulated by phosphorylation of tyrosine residues. One can, therefore, speculate that the variety of adaptor proteins that bind to these two regions within the cytoplasmic tail of β2 integrin differs at least in part compared with other β chains, and might determine why β2 is crucial for the formation of podosomes but not focal adhesion sites. In osteoclasts, podosomes – which organize into a dense ring called the sealing zone – still formed in the absence of β2 integrins. The reasons for this are not clear but the fact that β3 integrins are much more prominent in osteoclast podosomes versus DC podosomes may be relevant (supplementary material Fig. S2 and data not shown).

Our imaging studies in living DCs suggest that podosome-localized integrins are activated because their turnover was substantially slower than that of integrins in non-podosome regions of the membrane. This is consistent with the recent study of van den Dries et al., who showed that integrins in podosome plaques colocalize with talin (van den Dries et al., 2013b), and also an earlier publication demonstrating that – on T cells – the lateral mobility of integrin sub-populations is constrained when they are active and engaged with the cytoskeleton (Cairo et al., 2006). Our data support the model that has recently been proposed by van den Dries et al., whereby – in response to changes in tension – integrin–talin foci that are not already engaged with actin filaments await their recruitment to stabilize podosome actin cores when the need arises (van den Dries et al., 2013b). We have developed this picture by showing that the integrin array hosts sustained and continued formation, turnover and reformation of podosome cores, often at the same site that had been occupied by a previous core. This would allow core formation and tension sensing to be faster than if *de novo* priming and activation of integrins was required.

In immature DCs, podosomes are destabilized by TLR signaling although the kinetics and mechanistic basis appear to differ in murine versus human systems (van Helden et al., 2006; West et al., 2004). A key question only partially resolved in our study is why the presence of Ser745 and Ser756 is crucial for the TLR-signaled disassembly of podosomes. TLR-signaling did not measurably change the level of phosphorylation of these residues. The constitutive level of phosphorylation detected on Ser756 was clearly sub-stoichiometric because it could be increased ten times when the cells were treated with PMA. Nonetheless, positioning of a constitutively negative charge at these positions reduced integrin stability in podosomes. Ser745 is unique to β2 integrin and, so, may regulate β2-specific integrin properties. Phosphorylation of Ser745 is stimulated by outside-in signaling, for example following LFA-1 crosslinking (Perez et al., 2003). Ser756 is conserved in β1 integrin (numbered as residue 785 in β1 integrin) but not in β3 or β5 integrin. Destaing and colleagues have reported that, in fibroblasts, Src-induced invadosome formation is promoted by phosphorylation of β1 integrin at Ser785 or a constitutively negative charge at this position (Destaing et al., 2010), whereas another group found that the same modifications promote cell adhesion but inhibit cell migration (Mulrooney et al., 2001). The authors of the latter study suggested that this is due to weakened interactions between integrin and the cytoskeleton, which would be consistent with our data showing reduced persistence of Ser756Asp β2 integrin in podosome plaques. Generally, Ser/Thr phosphorylation, rather than Tyr phosphorylation, might play a more prominent regulatory role within the β2 integrin cytoplasmic tail, and has been shown to regulate interactions between integrin and its binding partners. For example, phosphorylation of Thr758 in the TTT region of β2 integrin promoted the binding of 14-3-3 proteins at the expense of filamin (Fagerholm et al., 2005). It will be important in future studies to determine whether additional specific binding partners are affected by Ser phosphorylation of β2 integrins in DCs and how, in conjunction with other TLR-signaled events, this promotes podosome breakdown. In connection to this, we have shown previously that activity of the protease ADAM17 is also required for TLR-signaled podosome dissolution in murine DCs (West et al., 2008), which emphasizes that β2 integrin phosphorylation alone is not a sufficient destabilizing stimulus and, indeed, β2 integrin carrying Ser756Asp or Ser745Asp mutations can still form podosomes, albeit at lower levels. The crucial substrate(s) of ADAM17 in this setting remain unknown. We looked for, but did not detect, an increase in β2 integrin shedding dependent on this protease in response to TLR signaling (M.A.W., unpublished). Mutations within the β2 integrin tail at Ser745 and Ser756 had no effect on PGE2-induced podosome destabilization in DCs, which is consistent with the distinct mechanism of podosome disassembly that involves enhanced myosin-II-based contractility, as described by van Helden and colleagues (van Helden et al., 2008). Thus, podosomes in DCs can be destabilized by, it seems, distinct pathways that involve either TLR-signaled perturbation of key integrin interactions or PGE2-driven perturbation of normal actomyosin contractility.

The findings reported here establish an experimental system that should permit further dissection of the role of integrins in podosomes. Combining high-resolution light microscopy and/or electron microscopy with improved antibodies against specific sites within the β2 integrin cytoplasmic tail may allow further investigation of the distinct integrin sub-populations – for example those with or without phosphorylated sites within their tail region – in the dynamic podosome arrays that are acutely regulated by immune stimuli in DCs.

## MATERIALS AND METHODS

### Reagents

Methanol-free paraformaldehyde (16%) was purchased from Agar Scientific (Stansted, UK) and lipopolysaccharide (LPS) and prostaglandin E2 from Alexis (Enzo Life Sciences Ltd, Exeter, UK). All other chemicals were from Sigma-Aldrich (Poole, Dorset, UK) unless stated otherwise.

### Mice and cells

Heterozygous embryos from *Itgb2-*null mice (Jackson Laboratories, ME; strain name: B6.129S7-Itgb2^tm2Bay^/J; Stock number: 003329) (Scharffetter-Kochanek et al., 1998) were implanted into stock C57BL/6 female mice. Offspring were genotyped as described on the Jackson Laboratories website. Bone marrow and spleen from *Itgb2*-null mice, β2 integrin TTT-AAA knock-in mice (Morrison et al., 2013) or from mice lacking expression αL, αM or αX integrin chains (provided by Nancy Hogg, Lincoln's Inn Field Research Institute, London, UK; Gordon Brown, University of Aberdeen, Scotland or Daniel C. Bullard, University of Alabama, AL, respectively) were used to generate BMDCs and SDCs as previously described (West et al., 2004; West et al., 1999). Lung cells were harvested by bronchoalveolar lavage with 1 ml PBS containing 5 mM EDTA and 2% FCS and collected by centrifugation at 300 ***g*** for 5 minutes at 4°C. The cells were resuspended in RPMI containing 2% FCS and differential cell counts performed on Diff-Quik-stained cytocentrifuge preparations (McMillan et al., 2002). All animal experiments were performed according to approved guidelines.

Osteoclasts were cultured from bone marrow according to Destaing et al. 2003 (Destaing et al., 2003). Briefly, cells (5×10^5^) were differentiated for 8 days on 13 mm glass coverslips in α-MEM (with 10% FCS) containing human M-CSF and sRANKL (both at 20 ng/ml; PeproTech EC Ltd, London, UK).

### Plasmids and constructs

Plasmids EGFP-N1, EGFP-C1, mCherry-N1 were from Clontech (Clontech Takara, Clontech Laboratories Inc., CA). The vector for retrovirus production, pBMN-I-GFP was provided by Gary Nolan (Stanford University, Stanford, CA). MiniRNA and Qiaex II gel extraction kits were purchased from Qiagen (Limberg, NL), and Accuscript First Strand cDNA Synthesis Kit, PCR-Script blunt-end cloning kit and QuikChange II XL site-directed mutagenesis kit from Stratagene (Agilent Technologies, UK). Quick Ligation Kit and restriction enzymes were from NEB (New England BioLabs, Herts, UK). Kits were used according to manufacturer's protocols.

Viral expression vectors containing murine Itgb2-EGFP, Lifeact-mCherry, EGFP-kindlin3 and paxillin-mCherry were constructed using standard cloning methods. Briefly, Itgb2 was amplified from spleen-derived DC (SDC) cDNA using primers 5′-ATGCTGGGCCCACACACTCACTGC-3′ and 5′-GCTTTCAGCAAACTTGGGGTTCATGACC-3′, then cloned into EGFP-N1 after introduction of *Eco*RI and *Sac*II restrictions sites. Site-directed mutagenesis was carried out by using this construct followed by insertion of the Itgb2-EGFP variants, using *Eco*RI and *Sac*II, into a modified pBMN-I-GFP plasmid from which GFP had been excised and replaced with a small cloning site containing *Mlu*I and *Sal*I restriction sites downstream of the IRES (pBMN-I).

A DNA fragment encoding the 17 amino acids of the F-actin-binding protein Lifeact (Riedl et al., 2008) was generated using primers 5′-TCGAGACGCGTACCATGGGCGTCGCTGACCTCATCAAGAAGTTCGAGTCCATCTCCAAGGAGGAGGCCC-3′ and 5′-GGG CCTCCTCCTTGGAGATGGACTCGAACTTCTTGATGAGGTCAGCGACGCCCATGGTACGCGT-3′, then subcloned into the *Xho*I and *Sma*I sites of mCherry-N1. Lifeact-mCherry was then inserted into pBMN-I using *Mlu*I and *Sal*I. To generate a bi-cistronic vector for co-expression of Itgb2-EGFP with Lifeact-mCherry, Itgb2-EGFP from Itgb2-EGFP-N1 was cloned into the pBMN-I-Lifeact-mCherry vector using *Eco*RI and *Not*I, to generate pBMN-Itgb2-EGFP-I-Lifeact-mCherry.

Mouse kindlin-3 was amplified from SDC cDNA using primers 5′-ATGGCGGGTATGAAGACAGCCTCC and 5′-TCAGAAGGCCTCATGGCCTCCTGTAAG, and cloned into EGFP-C1 using the *Eco*R1 and *Sal*I sites. *BamH*1 and *Sal*I were then used to clone EGFP-kindlin-3 into pBMN-I to generate pBMN-EGFP-kindlin-3. For the bi-cistronic plasmid pBMN-EGFP-kindlin-3-I-Lifeact-mCherry, EGFP-kindlin-3 was cloned into pBMN-I-Lifeact-mCherry using *Bam*HI and *Sac*II/*Stu*I (for blunt end cloning).

DNA for mouse paxillin was a gift from V. Verkhusha (Albert Einstein College of Medicine, Yeshiva University, NY; supplied by Addgene, MA; Plasmid no. 34964). This was fused to mCherry in mCherry-N1 using *Not*I and *Bam*HI, then *Stu*I and *Mlu*I sites introduced by PCR to allow cloning into pBMN-I to produce pBMN-paxillin-mCherry.

LZRSpBMN-EGFP-actin has been described previously (West et al., 2004).

### Virus production and cell infection

Retrovirus for protein expression in DCs was produced by transfection of the Phoenix Eco 293T packaging cell line (ATCC, LGC Standards, Middlesex, UK) with 15 µg of viral vector per 6-cm dish, as described (West et al., 2004). Virus containing supernatants were harvested 48 hours after transfection and used to infect BMDCs directly, or concentrated by mixing 3∶1 with lenti-X virus concentration solution (Clontech Takara), then centrifuging at 1500 ***g*** for 45 minutes at 4°C, followed by resuspension in 1/5th of the original volume. Infection of BMDCs was carried out on days 2 and 3 of culture, and cells used on day 7, as described (West et al., 2004).

### Immunofluorescence

Cells (2×10^5^) were plated in RPMI medium containing 10% FCS onto 13-mm glass coverslips (VWR International Ltd, Leicestershire, UK) for 4 hours at 37°C unless otherwise stated, then fixed with 4% paraformaldehyde in PBS for 15 minutes. The fixed cells were permeabilised for 3 minutes with 0.1% Triton X-100 in PBS, blocked with Image-iT FX signal enhancer (Life Technologies, Paisley, UK) then stained. F-actin was detected with Alexa-Fluor-555-conjugated phalloidin (Life Technologies), vinculin with mouse anti-vinculin (hVIN1, Sigma) and α-actinin with rabbit anti-α-actinin (Merck Millipore, MA). Integrins were stained with FITC-conjugated rat anti-mouse CD18 (β2, M18/2, eBioscience, Hatfield, UK), FITC-conjugated hamster anti-rat/mouse CD61 (β3, 2C9.G3, eBioscience) and rabbit anti-β5 (ab15459, Abcam, Cambridge, UK) followed by Alexa-Fluor-488-conjugated anti-rabbit IgG (Life Technologies). The cells were then mounted in Prolong Gold (Life Technologies) and left overnight to dry before being sealed onto slides. For quantitation of cells with podosomes, cells were assessed for the presence of at least one clearly identifiable podosome, with an F-actin-rich core and staining for a ring protein. At least 100 cells were scored per sample, with a minimum of three biological replicates.

### Flow cytometry

Wild type and Itgb2-null cells that had been virally infected or not were treated with 100 ng/ml LPS where indicated, preincubated with Fc block (2.4G2, BD Biosciences) then stained for surface expression of integrins or maturation markers using the following antibodies: CD11a (αL; 2D7, BD Biosciences), CD11b (αM; M1/70, eBioscience), CD11c (αX; HL3, BD Biosciences), CD29 (β1; Ha2/5, BD Biosciences), CD18 (β2; C71/16, Abcam), CD61 (β3; 2C9.G3, eBioscience), class II MHC (M5/114, eBioscience), CD40 (1C10, eBioscience), CD86 (Cambridge Bioscience, Cambridge, UK), CD54 (Cambridge Bioscience), CD274 (B7-H1; M1H5, eBioscience) and CD197 (CCR7; 4B12, eBioscience). Acquisition was performed using an LSR Fortessa flow cytometer (BD Biosciences). Data were analyzed using FlowJo software (TreeStar, OR), gating on cells expressing Itgb2-EGFP constructs where applicable.

### Extracellular matrix

Alexa-Fluor-488-conjugated gelatin and fibrinogen were from Life Technologies and FITC-conjugated hyaluronic acid (HA) from Sigma. Fibronectin (Life Technologies) and laminin (R&D Systems, Abingdon, UK) were conjugated to Alexa-Fluor-488 and Alexa-Fluor-555 fluorophores by using protein-labeling kits according to manufacturer's instructions (Life Technologies). Acid-washed coverslips were coated overnight with matrix components, then washed and blocked in RPMI with 10% FCS before plating with cells. The use of fluorescent labels allowed confirmation of the uniform distribution of the matrix components across the coverslip. Podosomes were labelled with Alexa-Fluor-555 or -633-phalloidin and anti-vinculin/Pacific Blue-rabbit anti-mouse IgG (Life Technologies).

### Adhesion assay

Flat-bottom 96-well Maxisorp plates (Nunc) were coated overnight at 4°C with ICAM-1, fibronectin or BSA (all at 6 µg/ml). Wells were then blocked with 1% BSA in phosphate-buffered saline (PBS) for 1 h at 37°C. The cells were resuspended in adhesion medium (RPMI with 0.1% BSA, 40 mM HEPES, 2 mM MgCl_2_) at 2×10^5^/ml before being added to blocked wells. The cells were allowed to adhere for 75 minutes at 37°C before unbound cells were removed by gentle washing in PBS/2 mM MgCl_2_. The bound cells were lysed in 1% Triton-X-100, 50 mM sodium acetate pH 5.0, containing 3 mg/ml p-nitrophenylphosphate (Calbiochem) and incubated for 1 h at 37°C in the dark; then the reaction was terminated by addition of 1M NaOH and the absorbance at 405 nm measured.

### Total internal reflection microscopy (TIRF)

DCs expressing fluorescence-tagged (EGFP and mCherry) constructs (pBMN-Itgb2-EGFP-I-Lifeact-mCherry and pBMN-EGFP-kindlin3-I-Lifeact-mCherry) were plated on 35-mm glass-bottomed dishes (FluoroDish, World Precision Instruments, Herts, UK) and imaged at 37°C using a Nikon Eclipse Ti TIRF microscope with perfect focus (Nikon UK Ltd, Surrey). Images were acquired every 10 seconds with an ApoTIRF 100x/NA1.49 objective and a 1.5x intermediate lens, using NIS-Elements AR 3.22 software and a Photometrics Cascade 10424 EMCCD camera. Kymographs were generated using Imaris software (Bitplane, Zurich, CH) and podosome lifetimes measured as described (West et al., 2008).

### Fluorescence recovery after photobleaching (FRAP)

DCs expressing fluorescent podosome components were plated on 35-mm glass dishes and imaged in a 37°C, 5% CO_2_ humidity chamber on a Zeiss LSM700 confocal microscope. Single optical 0.7 µm sections were acquired every 30 seconds (β2-integrin-EGFP) or every second (actin-EGFP, EGFP-kindlin3, paxillin-mCherry) before and after bleaching, by using a Plan Apochromat 100x/NA 1.46 objective. Bleaching was performed with 20 scans at 100% laser power. Recovery curves were plotted for each podosome component and fitted to the first rate order equation for determination of the half-lives of fluorescence recovery (Zen 2009 software, Zeiss).

### Mass spectrometric analysis of phosphorylated sites

BMDCs (7×10^6^) were plated in 10-cm dishes for 2 hours in RPMI containing 0.3% FCS, treated with LPS (100 ng/ml) or PMA (50 nM) for 25 min, before lysis for 15 min on ice with 1% NP-40 (Roche) in 50 mM Tris-HCl pH 7.5, 150 mM NaCl with 1 mM EGTA, 1 mM EDTA, 2 mM sodium orthovanadate, 25 mM sodium fluoride, 10 mM sodium β-glycerophosphate and protease inhibitors (Complete protease inhibitor cocktail, Roche) and centrifugation at 2000 ***g*** for 10 minutes. β2 integrin was immunoprecipitated for 2 hours at 4°C with 5 µg anti-β2 integrin (C71/16, Abcam), covalently coupled to protein G-Sepharose (Pharmacia) using dimethylpimelimidate (Harlow and Lane, 1988). Protein was eluted from the beads with 1% Rapigest (Waters Ltd, Hertfordshire, UK) and 5 mM TCEP (Pierce) in 50 mM Tris pH 8.0. Following alkylation with 10 mM iodoacetamide (quenched with 20 mM DTT) and 1∶10 dilution, proteins were digested with MS-grade trypsin (Pierce). After acidification and heating to cleave Rapigest, peptides were desalted by using solid-phase extraction. Mass spectrometric analyses were conducted on an Orbitrap Velos Pro coupled to an Ultimate 3000 UHPLC with a 50 cm EasySpray analytical column (Thermo-Fisher Scientific), using 2.5 hour linear gradients (solvent A: 3% DMSO, 0.1% formic acid, solvent B: 80% acetonitrile, 3% DMSO, 0.08% formic acid). Resulting data were compared against a Uniprot-Trembl *Mus musculus* database with Mascot 2.3 using Proteome Discoverer 1.4 and PhosphoRS 3.1 for qualitative and phospho analysis, and Andromeda/MaxQuant 1.4 for quantitative comparisons.

### Statistical analysis

Data are expressed as mean values, with error bars representing s.e.m (unless stated otherwise) from at least three biological replicates. Significance of differences between conditions was calculated by two-tailed paired or unpaired *t*-tests (Graphpad Prism 6, CA), and indicated by *P<0.05, **P<0.01, ***P<0.001 or n.s. (*P*>0.05).

## Supplementary Material

Supplementary Material
