# Supplementary material for: A crucial role for β2 integrins in podosome formation, dynamics and Toll-like-receptor-signaled disassembly in dendritic cells
Source: J Cell Sci. 2014 Oct 1;127(19):4213–24. doi: 10.1242/jcs.151167 (PMC4179490; doi:10.1242/jcs.151167)
Supplement: Supplementary Material [file supp_127_19_4213__index.html]

Supplementary Material 

# A crucial role for β2 integrins in podosome formation, dynamics and Toll-like-receptor-signaled disassembly in dendritic cells

## JCS151167 Supplementary Material

**Files in this Data Supplement:**

- **Supplementary Material**
