# Supplementary material for: A crucial role for β2 integrins in podosome formation, dynamics and Toll-like-receptor-signaled disassembly in dendritic cells
Source: J Cell Sci. 2014 Oct 1;127(19):4213–24. doi: 10.1242/jcs.151167 (PMC4179490; doi:10.1242/jcs.151167)
Supplement: Supplementary Material [file supp_jcs.151167_JCS151167.pdf]

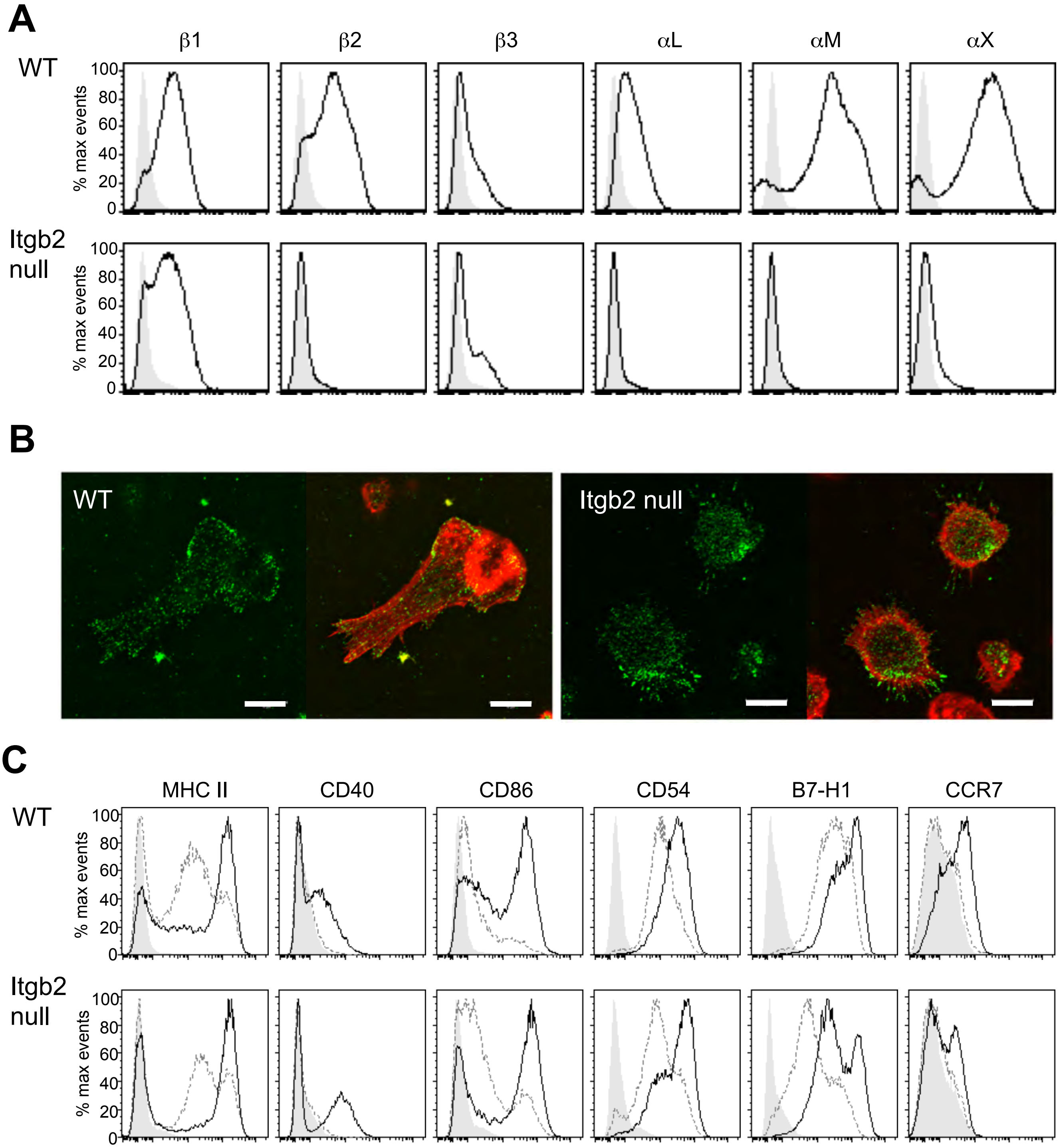

Fig. S1 Characterisation of Itgb2 null DC
WT and Itgb2 null BMDC were assessed for cell surface integrin expression by flow cytometry after staining for antibodies against β1, β2, β3, αL, αM and αX integrins (A). (B) WT and Itgb2 null DC were fixed and stained for β5 integrin (Alexa-555, green) and F-actin (Alexa-633, red) and imaged using a LSM700 confocal microscope as in Fig.1. Both WT and Itgb2 null DC express β5 integrin in focal adhesions. Scale bars, 10 μm. (C) To assess maturation, WT and Itgb2 null DC were left untreated (dashed lines) or treated with LPS (100ng/ml) for 20 hours (black line), stained for expression of surface [illegible]

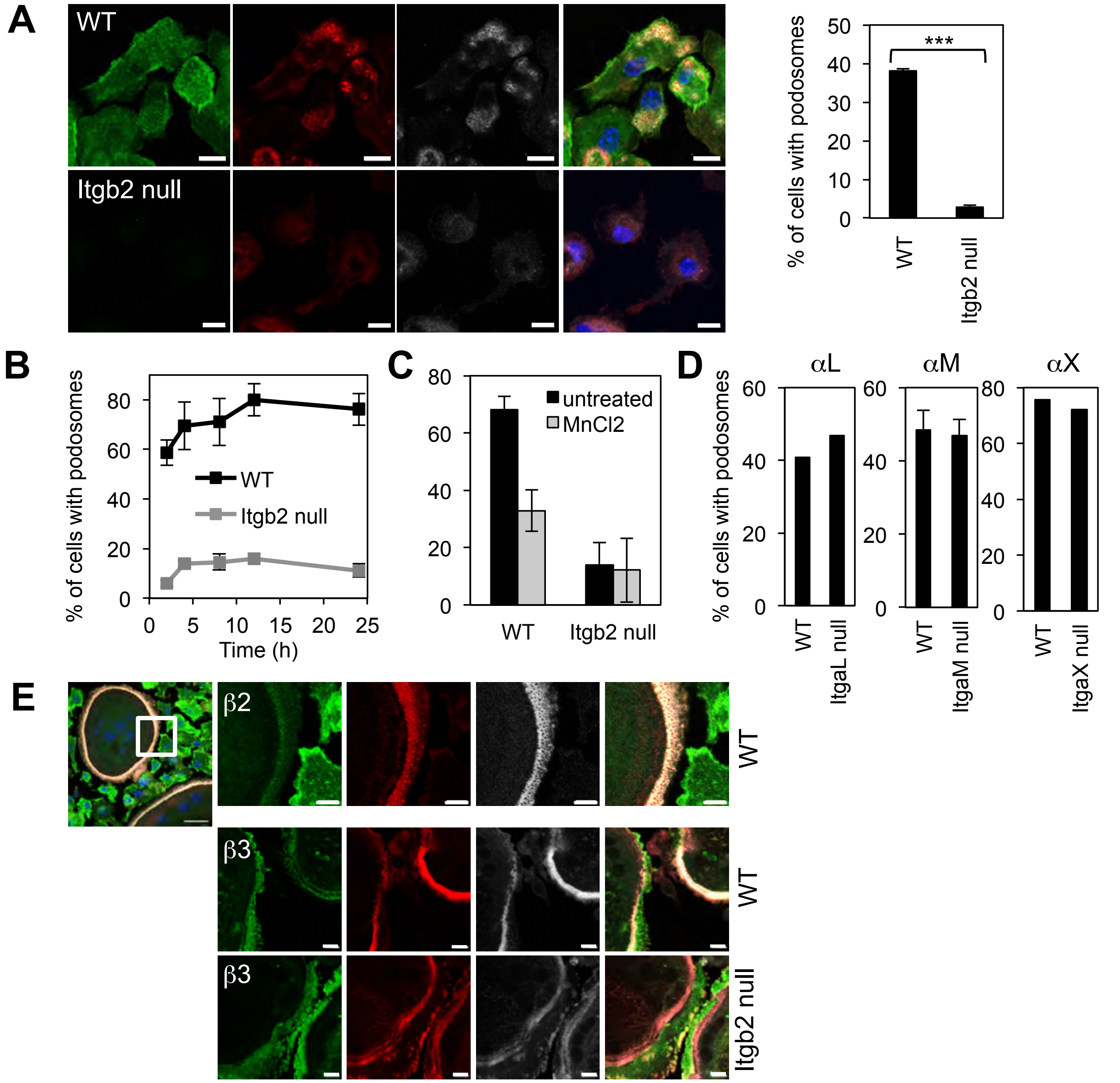

Fig. S2 A non-redundant requirement for β2 integrin in DC but not osteoclasts. (A) Wild type (WT) and Itgb2 null BMDC were stained for β2 integrin (FITC, green), F-actin (Alexa Fluor-555, red) and vinculin (Alexa Fluor-633, grey). Itgb2 null BMDC lack podosomes ($p < 0.0001$, unpaired t-test), as for SDC (Fig. 1). Scale bars, 10 μm. Podosomes were not rescued in Itgb2 null SDC allowed to adhere for extended times (B) or treated with 1 mM $MnCl_2$ for 30 minutes (C). (D) DC lacking αL (BMDC), αM (BMDC) or αX (SDC) integrin chains were assessed for podosomes. None of the 3 α integrin chains is individually required for normal podosome levels. (E) Osteoclasts from WT and Itgb2 null bone marrow were stained for β2 or β3 integrin (FITC, green), F-actin (Alexa Fluor-555, red) and vinculin (Alexa Fluor-633, grey). Integrin β2 in WT cells is found in the podosome ring around the podosome belt coincident with vinculin (top panels; magnified images of the boxed area). WT and Itgb2 null osteoclasts both express β3 integrins (middle and lower panels) which localise to podosome rings, observed even in the absence of β2 integrin. Scale bar, 20 μm (1st image) or 10 μm.

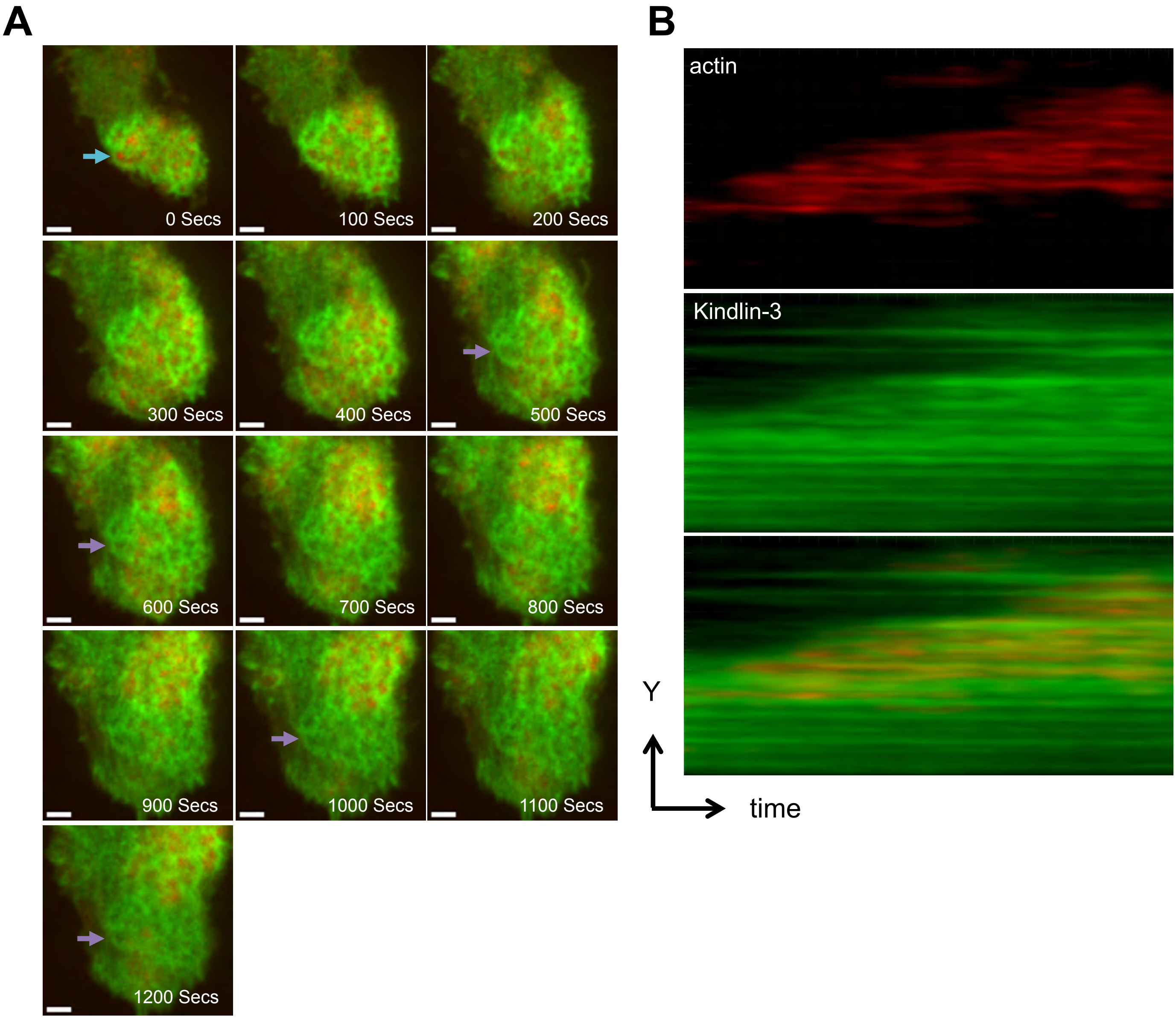

Fig. S3 Kindlin-3 dynamics in podosomes.
WT BMDC were infected with retrovirus for co-expression of EGFP-Kindlin-3 (green) and Lifeact-mCherry (actin, red). (A) TIRF images of a developing podosome plaque (micrographs taken every 100 seconds are shown) were acquired as in Fig. 3. The EGFP-Kindlin-3 persists in the podosome rings even after the majority of actin cores have left a given area. The blue arrow indicates a podosome ring in the periphery with an actin core present, whereas the purple arrows indicate the same region that remains persistent after the core has disappeared. Scale bars, 2 μm. (B) A TIRF sequence was used to generate a kymograph, as in Fig. 3 (Kindlin-3, green; actin, red; sequence represents 491 seconds), confirming the persistence of the kindlin labelled podosome rings compared with the transient nature of the actin cores. Slice in Y direction represents 26 μm.

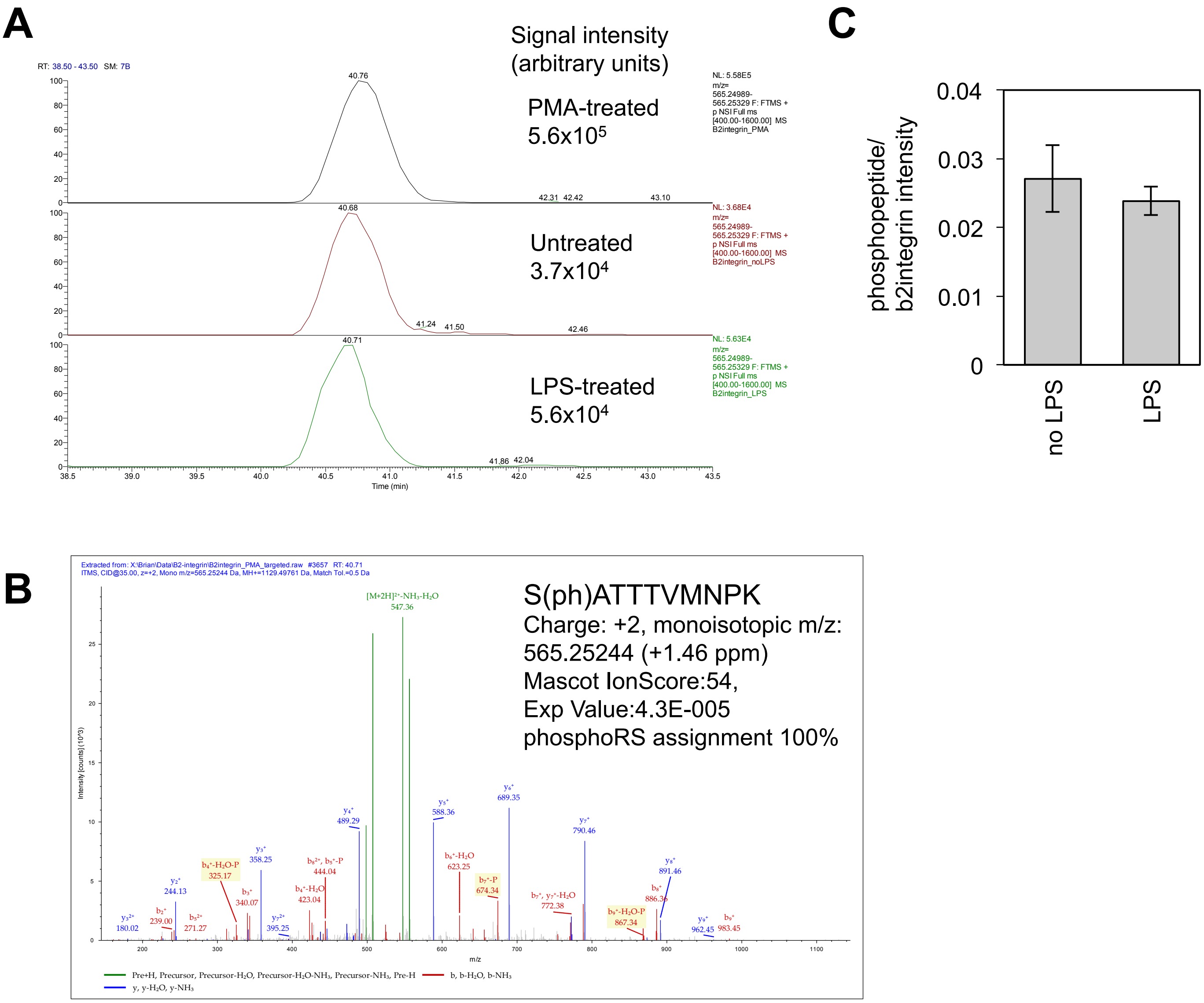

Fig. S4 The cytoplasmic tail of β2 integrin is phosphorylated at residue serine 756 in DC.

BMDC were allowed to adhere for 2 hours, then treated with LPS or PMA for 25 minutes or left untreated, before lysis and immunoprecipitation of total β2 integrin. Tryptic peptides from the eluted samples were subjected to LC-MS analysis for identification and quantitation of all peptides. (A) Extracted ion chromatogram of phosphorylated SATTTVMNPK shows relative abundance across IPs from PMA-treated, LPS-treated, or untreated DC. (B) Fragmentation spectrum for S(ph)ATTTVMNPK, with annotated ions demonstrating neutral loss ions. (C) Phosphopeptide intensities normalized to total β2 peptide intensities for each sample, calculated from MaxQuant LFQ intensities. Error bars indicate standard deviation based on three biological replicates.

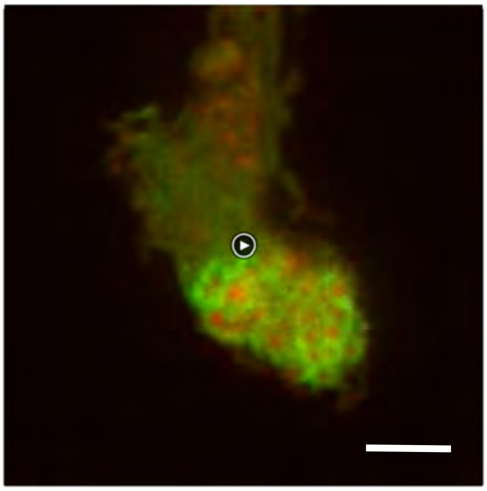

Movie 1. Kindlin and actin dynamics in podosome plaques in DC.
WT BMDC were infected with retrovirus to allow co-expression of EGFP-Kindlin-3 (green) and Lifeact-mCherry (actin, red) and live cell imaging carried using a Nikon Eclipse Ti TIRF microscope with an ApoTIRF 100x/NA1.49 objective as in Materials and Methods. EGFP-kindlin-3 in podosome rings is more persistent that the Lifeact-mCherry labelled actin cores. Images were collected every 10 seconds at 37°C. Movie shows 24.5 minutes of time-lapse imaging. Scale bar, 5μm.

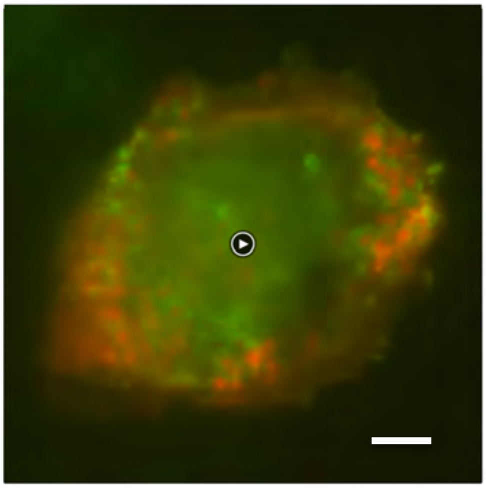

Movie 2. β2 integrin and actin dynamics in podosome plaques in DC.
Retrovirus encoding both Itgb2-EGFP and Lifeact-mCherry was used to infect Itgb2 null DC. The cells were imaged every 10 seconds at 37°C using a Nikon Eclipse Ti TIRF microscope with an ApoTIRF 100x/NA1.49 objective as in Materials and Methods. β2-EGFP labelled podosome rings are very stable compared to the actin cores, persisting after the cores have disassembled and in some cases hosting reformation of new cores. Movie shows 58.1 minutes of imaging. Scale bar, 5μm.
